# Health self-assessment by hemodialysis patients in the Brazilian Unified Health System

**DOI:** 10.1590/S1518-8787.2016050005885

**Published:** 2016-04-15

**Authors:** Tiago Ricardo Moreira, Luana Giatti, Cibele Comini Cesar, Eli Iola Gurgel Andrade, Francisco de Assis Acurcio, Mariângela Leal Cherchiglia

**Affiliations:** I Departamento de Medicina e Enfermagem. Universidade Federal de Viçosa. Viçosa, MG, Brasil; II Departamento de Nutrição Clínica e Social. Escola de Nutrição. Universidade Federal de Ouro Preto. Ouro Preto, MG, Brasil; III Departamento de Estatística. Instituto de Ciências Exatas. Universidade Federal de Minas Gerais. Belo Horizonte, MG, Brasil; IV Departamento de Medicina Preventiva e Social. Faculdade de Medicina. Universidade Federal de Minas Gerais. Belo Horizonte, MG, Brasil; V Departamento de Farmácia Social. Faculdade de Farmácia. Universidade Federal de Minas Gerais. Belo Horizonte, MG, Brasil

**Keywords:** Renal Insufficiency, Chronic, psychology, Renal Dialysis, Self-Assessment, Sickness Impact Profile, Attitude to Health, Outcome and Process Assessment (Health Care), Unified Health System, Cross-Sectional Studies

## Abstract

**OBJECTIVE:**

To examine whether the level of complexity of the services structure and sociodemographic and clinical characteristics of patients in hemodialysis are associated with the prevalence of poor health self-assessment.

**METHODS:**

In this cross-sectional study, we evaluated 1,621 patients with chronic terminal kidney disease on hemodialysis accompanied in 81 dialysis services in the Brazilian Unified Health System in 2007. Sampling was performed by conglomerate in two stages and a structured questionnaire was applied to participants. Multilevel multiple logistic regression was used for data analysis.

**RESULTS:**

The prevalence of poor health self-assessment was of 54.5%, and in multivariable analysis it was associated with the following variables: increasing age (OR = 1.02; 95%CI 1.01–1.02), separated or divorced marital status (OR = 0.62; 95%CI 0.34–0.88), having 12 years or more of study (OR = 0.51; 95%CI 0.37–0.71), spending more than 60 minutes in commuting between home and the dialysis service (OR = 1.80; 95%CI 1.29–2.51), having three or more self-referred diseases (OR = 2.20; 95%CI 1.33–3.62), and reporting some (OR = 2.17; 95%CI 1.66–2.84) or a lot of (OR = 2.74; 95%CI 2.04–3.68) trouble falling asleep. Individuals in treatment in dialysis services with the highest level of complexity in the structure presented less chance of performing a self-assessment of their health as bad (OR = 0.59; 95%CI 0.42–0.84).

**CONCLUSIONS:**

We showed poor health self-assessment is associated with age, years of formal education, marital status, home commuting time to the dialysis service, number of self-referred diseases, report of trouble sleeping, and also with the level of complexity of the structure of health services. Acknowledging these factors can contribute to the development of strategies to improve the health of patients in hemodialysis in the Brazilian Unified Health System.

## RESUMO

**OBJETIVO:**

Analisar se nível de complexidade de estrutura dos serviços e características sociodemográficas e clínicas de pacientes em hemodiálise estão associados à prevalência de autoavaliação de saúde ruim.

**MÉTODOS:**

Neste estudo transversal, foram avaliados 1.621 pacientes com doença renal crônica terminal em hemodiálise acompanhados em 81 serviços de diálise no Sistema Único de Saúde, no ano de 2007. A amostragem foi realizada por conglomerado em dois estágios e um questionário estruturado foi aplicado aos participantes. Para análise dos dados, foi usada regressão logística múltipla multinível.

**RESULTADOS:**

A prevalência de autoavaliação de saúde ruim foi de 54,5%, e na análise multivariada apresentou associação com as seguintes variáveis: aumento da idade (OR = 1,02; IC95% 1,01–1,02), estado civil separado ou divorciado (OR = 0,62; IC95% 0,34–0,88), ter doze anos ou mais de estudo (OR = 0,51; IC95% 0,37–0,71), gastar mais de 60 min no deslocamento entre a casa e o serviço de diálise (OR = 1.80; IC95% 1,29–2,51), apresentar três ou mais doenças autorreferidas (OR = 2,20; IC95% 1,33–3,62) e relatar alguma (OR = 2,17; IC95% 1,66–2,84) ou muita (OR = 2,74; IC95% 2,04–3,68) dificuldade para dormir. Indivíduos em tratamento nos serviços de diálise com maior nível de complexidade na estrutura apresentaram menor chance de autoavaliar sua saúde como ruim (OR = 0,59; IC95% 0,42–0,84).

**CONCLUSÕES:**

Autoavaliação de saúde ruim mostrou-se associada à idade, anos de estudo, estado civil, tempo de deslocamento de casa até o serviço de diálise, número de doenças autorreferidas, relato de dificuldade para dormir e também ao nível de complexidade da estrutura dos serviços de saúde. O reconhecimento desses fatores pode contribuir para o desenvolvimento de estratégias para melhorar a saúde dos pacientes em hemodiálise no Sistema Único de Saúde.

## INTRODUCTION

Health self-assessment is a comprehensive and solid subjective measure of health, besides being considered a strong predictor of disability^9^, usage of health services^14^, and mortality^7^. The context in which the individual lives can influence the health self-assessment, which, in turn, can aggregate and summarize the biological, psychosocial, and social dimensions of health^5^.

Among the factors associated with the worse health self-assessment are sociodemographic characteristics, such as female gender^4^, advanced age^4^ ^,^ ^19^, and lower levels of education^3^ ^,^ ^4^ and income^3^, as well as unhealthy behaviors^5^, presence of objective health conditions^19^, functional incapacity^3^ ^,^ ^4^ ^,^ ^20^, and psychosocial aspects, such as social isolation^5^. The presence of chronic diseases directly influences health self-assessment, and the presence of bad self-assessment grows as the number of referred diseases increases^4^.

Few studies have assessed the association between health self-assessment and chronic kidney disease^21^ ^,^ ^25^. Chronic kidney disease is a public health problem characterized by high morbidity and mortality, impacting economic, social, and personal aspects, as well as the health system. Patients with chronic kidney disease who develop end-stage renal disease require dialysis (hemodialysis or peritoneal dialysis) for the rest of their lives or require kidney transplant, carried out in specific health services^21^. The quality and the performance of the dialysis services are fundamental to maintaining the best clinical condition of the patients^27^. The characteristics of dialysis services influence the quality of life of patients in treatment^2^ and its results, as the reduction in the rate of urea after the hemodialysis session^23^, the control of anemia^8^ and the survival time^6^.

Studies have also shown that the prevalence of poor health self-assessment in hemodialysis patients with end-stage chronic kidney disease is high when compared to the prevalence observed in the total population^21^ ^,^ ^25^. In that case, similarities with the prevalence found in patients with other chronic diseases, such as coronary heart disease and cancer are pointed out^25^. This study aimed to assess whether the level of complexity of the structure of services and sociodemographic and clinical characteristics of patients in hemodialysis are associated with the prevalence of poor health self-assessment.

## METHODS

In this cross-sectional study, survey data were used by the Project TRS – Epidemiological and Economic Evaluation of Substitute Renal Therapy Modalities in Brazil, led by the Research Group in Health Economics at the Universidade Federal de Minas Gerais in 2007.

The TRS project participants were recruited in dialysis units (n = 81) and transplant centers (n = 17) registered at the Brazilian Society of Nephrology. The criteria for inclusion of participants were: being over 18 years old, being diagnosed with late-stage chronic kidney disease and being in dialysis for at least three months, or having performed a kidney transplant at least six months before. Patients who did multiple transplants were excluded.

A representative sample was obtained at the services of dialysis and transplant centers contracted with the Brazilian Unified Health System (SUS). Sampling by conglomerate was used in two stages: in the first one, dialysis services and transplant centers were sampled; in the second, patients in treatment at dialysis centers and transplant services sampled in the first stage were selected. The random selection of patients was made using the records of patients of dialysis services and transplant centers. We selected 3,036 patients, of which 1,621 were in hemodialysis, 788 in peritoneal dialysis, and 627 who had performed kidney transplant. Each sample is representative of the respective treatment modality.

Data collection was carried out from January to May 2007. Trained interviewers applied a previously tested structured questionnaire containing socioeconomic, demographic, clinical, and quality of life questions. The patients were given a copy of the questionnaire to follow during the implementation of the interview. Only patients who were undergoing hemodialysis treatment were assessed, which corresponded to 1,621 individuals allocated in 81 dialysis services in the Country. The average number of patients by dialysis service was of 20 (minimum of 13 and a maximum of 31 patients).

Dialysis services data analyzed were obtained in the National Registry of Health Establishments (CNES)^a^, made available through DATASUS, Ministry of Health. The data obtained concern the same period of data collection from patients.

The dependent variable was the health self-assessment. The answers to the question “how would you say your health is in general?” were categorized as “good” (which included individuals who answered excellent, very good or good), and “bad” (which included respondents who answered regular or bad).

Explanatory variables were included to the individual (level 1) and dialysis services (level 2). The sociodemographic characteristics investigated were: sex, age (continuous and grouped into age groups), color or race, full years of education, marital status, and commuting time between home and the dialysis service. The clinical characteristics were: time on dialysis, number of self-referred diseases (constructed from the responses to the questions that investigated medical diagnosis of hypertension, diabetes, cancer, depression, pulmonary disease, cirrhosis, arthritis, HIV, bone disease, stroke, ulcers, and hepatitis), inscription on the transplant list, trouble sleeping, extra medical consultation (out of the dialysis service) in the last 12 months, and hospitalization in the last 12 months.

Contextual characteristics related to dialysis service were: type of service (outpatient; inpatient), educational activity (yes; no), number of hemodialysis machines (2-20; 21-30; 31 or more), working shifts (morning and afternoon; morning, afternoon, and night; 24-hour), existence of transplant service in the unit (yes; no), existence of vascular surgery service (yes; no), and dialysis treatments offered (hemodialysis; hemodialysis and peritoneal dialysis). These variables were grouped into an indicator that characterized the level of complexity of dialysis service structure through the analysis of the main component (AMC) from the polychoric matrix correlation^b^. This analysis reduced the number of variables assessed, generating a new set of p components, with the first component being the one that presents the greatest variance possible among all possible linear combinations of the original variables. The extracted component is a continuous variable categorized according to tertiles, defining the level of complexity of the dialysis services in high, medium, and low. The component obtained explained 72.0% of the total variance of the seven variables used in characterizing the structure of the dialysis services.

The initial analysis included description of study population by means of frequency distribution, mean, and standard deviation. The prevalence of poor health self-assessment was estimated, and its association with characteristics of the individual and the service was investigated using Pearson’s Chi-square test with a significance level of 5%. The strength of the association between health and self-assessment explanatory variables was evaluated by odds ratio (OR) and their respective 95% confidence intervals using bivariate and multivariate multilevel logistic regression.

Mean odds ratio (MOR) was used to estimate the contextual effect. MOR quantifies the variation among the dialysis services, comparing two people with the same covariables in two services picked randomly^13^. MOR is the median chances ratio between the individual that is more likely and the one that is less likely to report poor health self-assessment. As MOR quantifies the variance of the contextual level in the odds ratio, it is comparable to the fixed effects odds ratio. Thus, MOR measures heterogeneity in scale familiar to researchers who worked with the logistics regression model^13^.

The association between each variable of level 1 and the self-perception of health was tested (bivariate analysis). The following multilevel modeling was started with the setting of a null model (without explanatory variables) to verify the significance of variance of the poor health self-assessment among dialysis services (model I). The subsequent model (model II) was adjusted with the inclusion of explanatory variables of individual level that presented value of p < 0.20 in bivariate analysis. The ones with p-value < 0.05 were kept. The final model (model III) included the contextual level variable, complexity of dialysis service structure to the previous model, with the variables that showed statistical significance at the level of 5% remaining.

We used Akaike Information Criterion (AIC) and likelihood ratio test to compare the models. The Stata software version 11.0 was used for statistical analysis.

The TRS Project was approved by the Research Ethics Committee of the Universidade Federal de Minas Gerais (Opinion ETIC 397-04). All participants signed an informed consent form.

## RESULTS

Of the 1,621 participants, more than half were male, married, and with up to seven years of study. The average age was 48.9 years old (SD = 14.5) and approximately 76.0% were less than 59 years old. More than a third was white or yellow and about 33.0% reported spending less than 20 minutes on the way home to the dialysis service. Other features of interest are found in Table 1.

**Table 1:** Sociodemographic characteristics, clinics and dialysis services to patients on hemodialysis. Brazil, 2007.

Variable	n	%
Health Self-assessment		
Good	738	45.5
Poor	883	54.5
Sociodemographic features		
Sex		
Female	707	43.6
Male	914	56.4
Age group (years old)		
18-39	446	27.5
40-59	781	48.2
60 or more	394	24.3
Color or race		
White and yellow	671	41.9
Black	230	14.3
Brown	588	36.7
Others	114	7.1
Marital status		
Married	921	56.8
Single	385	23.8
Separated/Divorced	182	11.2
Widower	130	8.0
No information	3	0.2
Completed education in years		
0-3	337	20.8
4-7	567	35.0
8-11	248	15.3
12 or more	465	28.7
No information	4	0.2
Time spent between home and the dialysis service		
< 20 min	543	33.5
20-40 min	464	28.6
41-60 min	313	19.3
> 60 min	301	18.6
Clinical features		
Time in dialysis		
< 6 months	141	10.3
7-12 months	192	14.0
13-24 months	283	20.7
< 24 months	752	55.0
Registry in the transplant waiting list		
Yes	903	55.7
No	656	40.5
Additional medical consultation		
No	740	45.7
Yes	880	54.3
No information	1	0.1
Hospitalization in the last 12 months		
No	869	53.6
Yes	747	46.1
No information	5	0.3
Trouble falling asleep		
None or a little amount of time	856	52.8
Some or a good amount of time	401	24.7
Most of the time	364	22.5
Self-referred diseases		
None	100	6.2
One	541	33.4
Two	492	30.4
Three or more	455	28.1
No information	33	2.0
Characteristics of the dialysis services		
Complexity level of the dialysis service structure		
Low	553	34.1
Average	535	33.0
High	533	32.9

Total	1,621	100

Among the participants, 54.5% reported poor health self-assessment (Table 1). In bivariate analysis, the chance of poor health self-assessment increased with age, and was highest among those who spent more than 60 min on the way home to the dialysis service than those who spent less than 20 min. The prevalence was lowest among single, separated, and divorced patients than married ones, and lowest among those who studied eight years or more compared to those who reported up to three years of study. With the exception of time on dialysis treatment, all clinical characteristics were associated with poor health self-assessment (p < 0.05). In addition, the chance of poor health self-assessment was lower in patients attended in services of high complexity level (OR = 0.67; 95%CI 0.53–0.86) (Table 2).

**Table 2:** Gross and adjusted analysis of multilevel logistic regression for factors associated with poor health self-assessment of hemodialysis patients. Brazil, 2007.

Individual-level variables	Gross analysis		Adjusted analysis				
	
	OR	95%CI	Model I	Model II		Model III	
		
				OR	95%CI	OR	95%CI
Sociodemographic features							
Sex							
Female	1	-	-	-	-	-	-
Male	1.01	0.82–1.23	-	-	-	-	-
Age	1.02	1.01–1.03*	-	1.02	1.01–1.02*	1.02	1.01–1.02*
Color or race							
White and yellow	1	-	-	-	-	-	-
Black	1.11	0.81–1.52	-	-	-	-	-
Brown	1.22	0.97–1.55	-	-	-	-	-
Others	1.20	0.79–1.82	-	-	-	-	-
Marital status							
Married	1		-	1		1	
Single	0.78	0.60–0.99*	-	1.15	0.86–1.54	1.14	0.86–1.53
Separated/Divorced	0.68	0.49–0.95*	-	0.63	0.44–0.89*	0.62	0.43–0.88*
Widower	1.22	0.83–1.79	-	0.89	0.57–1.38	0.90	0.58–1.38
Completed education in years							
0-3	1	-	-	1		1	-
4-7	0.86	0.65–1.15	-	0.96	0.70–1.31	0.96	0.71–1.31
8-11	0.69	0.49–0.97*	-	0.80	0.54–1.17	0.80	0.55–1.17
12 or more	0.44	0.32–0.59*	-	0.51	0.36–0.71*	0.51	0.37–0.71*
Time spent between home and the dialysis service							
< 20 min	1	-	-	1	-	1	-
20-40 min	1.13	0.87–1.48	-	1.05	0.79–1.39	1.06	0.80–1.40
41-60 min	1.22	0.91–1.63	-	1.14	0.83–1.57	1.17	0.85–1.60
> 60 min	1.83	1.35–2.48*	-	1.74	1.25–2.43*	1.80	1.29–2.51*
Clinical features							
Time in dialysis							
< 6 months	1	-		-	-	-	-
7-12 months	0.86	0.55–1.35	-	-	-	-	-
13-24 months	0.88	0.58–1.35	-	-	-	-	-
> 24 months	0.87	0.59–1.27	-	-	-	-	-
Registry in the transplant waiting list							
Yes	1	-	-	-	-	-	-
No	1.33	1.07–1.65*	-	-	-	-	-
Additional medical consultation							
No	1		-	1	-	1	-
Yes	1.36	1.11–1.67*	-	1.27	1.01–1.60*	1.24	0.99–1.57
Hospitalization in the last 12 months							
No	1	-	-	1	-	1	-
Yes	1.41	1.15–1.74*	-	1.24	0.99–1.55	1.25	1.00–1.57
Trouble falling asleep							
None or a little amount of time	1	-	-	1	-	1	-
Some or a good amount of time	2.31	1.79–2.98*	-	2.16	1.65–2.83*	2.17	1.66–2.84*
Most of the time	3.25	2.47–4.28*	-	2.68	2.00–3.61*	2.74	2.04–3.68*
Self-referred diseases							
None	1	-	-	1	-	1	-
One	1.36	0.86–2.13	-	1.33	0.83–2.15	1.28	0.80–2.07
Two	1.88	1.19–2.98*	-	1.55	0.95–2.53	1.47	0.90–2.39
Three or more	3.08	1.92–4.91*	-	2.33	1.41–3.84*	2.20	1.33–3.62*
Context-level variable							
Level of complexity of the dialysis service structure							
Low	1	-	-	-	-	1	-
Average	1.01	0.73–1.37	-	-	-	0.94	0.69–1.33
High	0.67	0.49–0.91*	-	-	-	0.59	0.42–0.84*
Measuring the variation among services							
σ^2^ (EP)	-	-	0.411 (0.076)	0.479 (0.081)		0.412 (0.083)	
PVC	-	-	-	16.7%		-14.0%	
MOR	-	-	1.48	1.58		1.48	
Evaluation of the Models	-	-					
Log likelihood	-	-	-1108.966	-986.0015		-981.0539	
Test LR	-	-	-	< 0.001		0.007	
AIC	-	-	2221.931	2010.003		2004.108	

OR: odds ratio; σ^2^: variance in the context level; EP: standard error; PCV: proportional variance change; MOR: median odds ratio; LR test: likelihood ratio test; AIC: Akaike information criterion

* p < 0.05.

The adjusted multilevel analysis is presented in Table 2. Model I presents a statistically significant variance in self-assessment of poor health among dialysis services (σ^2^ = 0.411; 95%CI 0.285–0.591). MOR was equal to 1.48, indicating that differences between dialysis services may increase in 48.0% the odds of poor health self-assessment. For instance, if the patient can transfer to a dialysis service with more inadequate service conditions, the risk of self-evaluating his health as bad could increase, being the median of such increase equal to 48.0%.

In model II, the following individual variables remained independently associated with increased chance of poor health self-assessment: being older, spending 60 minutes or more on the way home to the dialysis service, having three or more of the diseases referred, and having trouble sleeping. Separated or divorced marital status, and having 12 years or more of study were associated with less chance of poor health self-assessment.

As to the contextual level (level 2), the chance to assess health as poor was lower among those who were under treatment in dialysis services with the highest level of complexity (OR = 0.59; 95%CI 0.42-0.84) (model III). The inclusion of context variable did not cause major changes in the magnitude of the association between poor health self-assessment and individual variables. Only the variable “extra medical consultation” (out of dialysis service) lost its significance. The variance of poor health self-assessment among dialysis services was reduced from 0.479 (model II) to 0.412 (model III) after the inclusion of the context variable, indicating that the variation of 14.0% of poor health self-assessment in the contextual level might be explained by the inclusion of the level of complexity of the dialysis service structure in the model. A reduction of AIC between models II and III occurred, and the likelihood ratio test showed to be significant (p < 0.05), indicating better adaptation of model III.

## DISCUSSION

The results of this study show that more than half of the patients in hemodialysis assessed their health as poor. Patients who presented a higher chance to assess their health negatively were the older, less-educated ones, who spent more time on the way home to the dialysis service, which reported the largest number of self-referred diseases, and difficulty sleeping. Separated or divorced patients had less chance of poor health self-assessment. Our results showed that those who were on hemodialysis services characterized by increased complexity of structure presented a chance to assess their own health negatively.

The prevalence of self-assessment of poor health is extremely high compared to the one observed in the adult population that resided in households covered by the fixed phone network in the Brazilian state capitals and the Federal District in 2012, which was of 5.0%^c^. However, it is similar to that found among patients on dialysis in Denmark^21^ and Holland^25^, respectively 51.0% and 54.0%. It is also similar to the percentages of adults who reported having any long-term disease or disability in Brazil in 2003, 55.9%^24^, and in the elderly from different Brazilian cities in 2009, approximately 50.0%^20^. However, the population of this study is younger than the population of the studies cited. Such high prevalence of poor health self-assessment found in hemodialysis patients draws attention, since many authors have noted the high predictive power of morbidity and mortality resulting from a poor assessment of their own health^7^.

The results were consistent with population-based studies with adults^4^ and older adults^20^ being influenced by age, education, and the number of self-referred diseases in the health self-assessment. An association between self-assessment of poor health and sex was not identified, unlike other studies in adults^10^ and older adults^3^ ^,^ ^4^. The chance to assess health as bad was higher among individuals with lower education levels, regardless of other characteristics investigated. Education determines several advantages for health, because individuals with higher educational level are less likely to expose themselves to risk factors for diseases and, in general, have greater access to economic and social resources that promote better health^18^.

The association between the presence of diseases and poor health self-assessment confirms the account of other authors who have evaluated that relationship in surveys with adults in Brazil^4^ ^,^ ^c^. Nonetheless, in this population under the impact of a more severe chronic disease, which evolves with complications and restrictions in daily activities, only the account of three or more diseases were associated with poor health self-assessment. Other studies also identified the association between multiple morbidities and worse health assessment^17^. Other frequent disorders among these patients, such as changes in sleep, are related to the poor health self-assessment, as shown by our results. Similar association was also reported in hemodialysis patients in Italy^16^. Sleep disorders are associated with increased cardiovascular morbidity and mortality in the general population. As the incidence of these disorders is considerably higher among patients in end-stage chronic kidney disease, the potential effects could also be higher in this population^26^.

In this study, separated or divorced marital status was associated with less chance of reporting poor health self-assessment. A research carried out in Italy with hemodialysis patients showed that living alone or with other people has no association with poor health self-assessment^16^. However, studies in adults and older adults in Sweden^11^ and Japan^10^ showed that poor health self-assessment was associated with, respectively, being single and being divorced. In fact, the social support promoted by a partner or by family members is one of the most important factors to face the disease and treatment, since it contributes to a reduction in the incidence of depression and mortality, and to improve the quality of life of patient undergoing hemodialysis^1^.

The use of dialysis service is part of the weekly routine of these patients. The commuting time to the dialysis service can affect the health self-assessment of the patient, since it adds to the time that patients spend on dialysis. In this study, spending a longer time getting to the dialysis facility was associated with poor health self-assessment. A study performed with a representative sample of patients in hemodialysis of 12 countries found a higher risk of death and significantly lower quality of life in patients with longer commuting times to the dialysis service facility^15^. The longest time spent in the way to the service decreases the time available for other activities, also infuencing patients’ sleep and satisfaction with the received care^19^.

This is the first national-level study to investigate the prevalence of self-assessment of poor health in hemodialysis patients and its association with individual characteristics and dialysis services. It was possible to identify significant variance in poor health self-assessment among dialysis services after the control by sociodemographic and clinical variables. An increase in variance of poor health self-assessment was noticed among dialysis services in model II. This can happen when individual-level variables are negatively correlated with variation in the contextual level. The variance in poor health self-assessment among dialysis services in model II indicates the true variance between the services, after adjusting according to the differences in the profile of patients assisted in each unit.

The level of complexity of the service structure can explain 14.0% of variability in prevalence of poor health self-assessment among hemodialysis services, pointing to the importance of the structure of services for the maintenance of better health condition of these patients.

Individuals attended in dialysis services with better structure presented a lower chance to assess their health as poor. The highest level of complexity of dialysis service structure was characterized by location in hospitals, having a teaching activity, having the highest number of dialysis machines, working in three daily shifts or 24h, having its own vascular surgery service, performing kidney transplant, and performing peritoneal dialysis and hemodialysis. It is possible that the patients in services with more complex structure have greater probability of access to alternative treatment in the same service, such as implementation of arteriovenous fistula, peritoneal dialysis, and transplant, which may influence the perception of security and better care from patients.

Other studies also identified a significant association between the structure of dialysis services and some results on patients’ health. Carvalho et al.^6^ found better results on survival of patients attended in dialysis services with the highest number of dialysis machines. In the same study, patients submitted to hemodialysis in services where cyclical peritoneal dialysis was the only treatment option had lower survival compared to those where there was no peritoneal dialysis service. Machado et al.^12^ noted that the access to kidney transplant was greater in dialysis services with less complex structure^12^. New studies may delve into aspects of how the structure of the services influence the self-assessment of health of patients in hemodialysis treatment.

Another possible explanation for the variability of poor health self-assessment among dialysis services can be attributed to differences in care practices. A study performed on 54 dialysis services in Italy showed that the greater social support performed by health professionals in dialysis services was associated with better health self-assessment^16^. Spiegel et al^22^ identified lower rates of mortality in dialysis services in which there were interpersonal relationships, more communication with the doctor, and earlier multidisciplinary meetings to discuss strategies for treatment after hospital discharge. In that case, the nutritionists addressed cultural aspects relevant to the patients’ care plan, better quality continuing education programs, and there was more engagement and discipline from the patients. Such differences have explained 31.0% of the variation of rates of mortality among the 90 dialysis services in the United States participating in the study in 2007^22^.

Among the limitations of this study, we can cite the indicator used. Although there are many advantages in the use of health self-assessment as an indicator of health, limitations are described in literature about the lack of knowledge of the mechanisms and ways with which each individual evaluates their own health^7^. Another limitation refers to the use of the administrative registries of the CNES to characterize dialysis services. The use of such data, whose record does not aim to be used in research activities, can have the occurrence of incompleteness and inconsistencies as a limitation. Other variables that characterized the service, such as number of rooms and number of dialysis professionals were not included in the analysis due to the great loss of information. The last limitation is inherent to the design, which does not allow to state if the associated factor is determinant or determined by poor health self-assessment.

In conclusion, the high prevalence of poor health self-assessment found among hemodialysis patients in the SUS system was associated with age, years of education, marital status, commuting time from home to the dialysis service, number of self-referred diseases, and reports on difficulty of sleeping. Health self-assessment in this population does not involve only individual factors, but it is also related to the level of complexity of the structure of health services, confirming the multidimensional nature of this health indicator.

The acknowledgment of the effects of dialysis services in poor health self-assessment draws attention to the importance of the structure of the facilities, as an additional dimension of the strategy to improve the health of patients in hemodialysis in SUS. It is necessary that health professionals are alert to the aspects linked to worse health self-assessment and that public policies are implemented to prevent or delay undesirable forecasts in this population.

## Footnotes

a Ministério da Saúde. Cadastro Nacional de Estabelecimentos de Saúde – CNESNet. Brasília (DF); 2007 [cited 2007 Jan 15]. Available from: http://cnes2.datasus.gov.br/Listar_Unidade_Dialise.asp

b Kolenikov S, Angeles G. The use of discrete data in principal component analysis with applications to socioeconomic indices. Chapel Hill: University of North Carolina, Carolina Population Center; 2004. (CPC/MEASURE Working paper nº WP-04-850).

c Ministério da Saúde, Secretaria de Vigilância em Saúde, Departamento de Vigilância de Doenças e Agravos não Transmissíveis e Promoção de Saúde. Vigitel Brasil 2012: vigilância de fatores de risco e proteção para doenças crônicas por inquérito telefônico. Brasília (DF); 2013.
